# Homœopathy in Italy

**Published:** 1845-08

**Authors:** Edwin Lee


### SELECTIONS

FROM

### AMERICAN AND FOREIGN JOURNALS.

---

*Homœopathy in Italy.* By Edwin Lee, M. D.—Homœopathy has long ago been judged in France. Science, this time in accordance with public opinion, has condemned it without appeal. It is dead, absolutely dead, notwithstanding some useless attempts at galvanizing after the blow. Its existence was there most ephemeral; scarcely are any of its proselytes now to be found in Paris, and hardly half a dozen in the provinces.

There are some minds which seem naturally to be seized with a vertigo in the presence of novelty, or of an extraordinary unheard-of fact. Every new thing assumes in their eyes the color of truth; their good sense succumbs before every thing which is invested with an original character. This disposition depends upon the exaggeration of a legitimate sentiment; for who at the present day is not an advocate for the progress of science? who would dare, for instance, to pretend that medicine has reached its utmost limits, and who would wish to retain it within the bounds of ancient traditions? But wise and prudent men are careful, before entering upon a new path, to watch its direction, and to estimate its solidity. When they give a judgment it is with reserve, and with circumspection. This has appeared to us to be the only rational position to take with respect to homœopathy. After having studied it in its books, its journals; after having assisted at its experimentation at the bed-side of patients, and in hospitals; we concluded by pronouncing that Hahnemann is no more than the missionary of a paradoxical idea, destroyed *à posteriori* by observation, ruined by examination, and of which all the value is reduced to a last blow at the doctrine of Broussais.

Homœopathy made itself a favorable argument in Italy, of its pretended universal application in France, which was the counterpart of its representations in Paris, where it proclaimed the whole of Italy to be converted to its dogmas; that the sovereigns and population of the peninsula could not dispense with the help of its new materia medica; and that even teaching was imbued with its principles. In the presence of such facts, asserted with so much audacity, we deemed it requisite to follow up our former investigations, and to submit the doctrine to a new judgment, inasmuch as we might suppose it to be better appreciated elsewhere than in our own country. But we have had the demonstration of the falsehood of these assertions, which were doubtless unknown to the official propagators of the new German theory.

And, in the first place, what is now to be understood as homœopathy? It is, perhaps, the sentiment of Hahnemann literally expressed in his writings, free from any amalgamation of the medicine of his opponents, which he so strongly denounces. In fact, in his eyes ancient and modern practitioners represent so many homicides, armed with diplomas sanctioned by the laws, exercising with impunity a fatal profession. Is it that exclusive theory, based upon so minute and difficult a symptomatology, with its infinitesimal and almost impalpable doses, possessing, nevertheless, a miraculous activity? Or is it this same doctrine modified, more tolerant, less forgetful of the scientific genealogy, of which its adepts remember to have seen formerly some real and efficacious effects, which will neither give up bleeding in appoplexy and in pneumonia, nor sulphate of quinine in large doses in intermittent fevers? The few disciples of Hahnemann in France now adopt these mixed ideas; they believe in this medical *juste-milieu.*

Well, whichever of them be meant, Italy rejects at the same time all these gradations; it neither believes in the genius nor in the wonders of Hahnemann, notwithstanding a natural inclination for all that comes from Germany. At Milan, homœopathy constitutes the practice of two or three Austrian physicians. At Lucca, one of its followers is patronized by the Duke, who, however, likewise keeps near him a very orthodox allopathic physician.

We were for a long time curious to know the opinion of Tommassini, this patriarch of Italian medicine, with regard to homœopathy; and the more especially as they arrive at conclusions diametrically opposed—as far as the poles asunder—the one prescribing infinitesimal doses, and the other enormous quantities of remedial substances, even in the opinion of

French physicians. We, therefore, took an opportunity of asking the Professor of Parma, what was, in his eyes, the medical value of homœopathy. To this inquiry he first of all stated that the same question had been proposed to him in the same terms, in a meeting of the Royal Academy of Naples, and that he had refused to express his opinion in public, inasmuch as he had made no trials of the doctrine; but that at a later period, after having made it the subject of serious examination, he had frankly expressed himself in a discourse to his pupils while residing at Bologna.

"I do not conceive," said he, "that a method sometimes innocent, but often dangerous can be adopted. I can understand that, in chronic diseases, in complaints where the beginning of a new treatment can be postponed to the following spring, in which one amuses the patient instead of subjecting him to a curative method, homœopathy is exempt from serious inconveniences—as, for instance, in asthma and other diseases of the same nature; but it does not do in this manner when acute affections are under consideration—pneumonia, enteritis, or when an organ is threatened with gangrene, in which, from Hippocrates down to the present day, energetic remedies have been with reason recommended—bleeding, leeches, purgatives, &c. I would then mistrust a doctrine which has no regular origin, and refuses all paternity."

If anything more were required to corroborate so explicit a condemnation, we would add that the Italian youth, so animated, ardent and eager after fresh knowledge, and withal liberal enough not to receive, without examination, opinions already formed, to whom both the present and future belong, without being burthened with doctrines and practices more or less ancient, did not receive the doctrine of Hahnemann in a more favorable manner than others. We have seen many students and many young professors, but we have not known one who had declared himself his follower.

The population also disdained the numerous benefits which it was pretended they would enjoy, and homœopathy was not in this respect more successful in the peninsula than in France. Its course may be likened to that of the practice which thought to ascertain all diseases by means of an inspection of the urine—and to that of all universal panaceas which appear, and die upon being subjected to trial, as, for instance, the hydro-sudopathy, to which a premature end may be predicted. Nevertheless, it must be confessed, that Hahnemann's theory rests upon a more dogmatical principle, perhaps, because its author lived a long time in Germany, a land so erudite, so abounding in synthetical ideas, and evidently for the reason that it arises from the vitalist theory, which is the daughter of the Stahlian *animism.* On the other hand, we must not foget, that for its part it was fortunate in its criticisms directed against the anatomo-pathologists, whose material conceptions were concentrated to so circumscribed and exclusive a point of view.

In order to omit nothing that may tend to elevate the progressive element of homœopathy, it must likewise be affirmed that by its means allopathy has been brought again to the study of the moral part of man; which study has been much neglected since the close of the eighteenth century. Nevertheless, the medicine of the mind is no less important than that of the body; or, to express the idea better, science should never separate the one from the other in their reciprocal and harmonical relations.

In conclusion, homœopathy has neither been able to introduce itself in Italy, in instruction, nor among the true public (by which is meant the most numerous class); accident cast it, in the first instance into the centre of some courts. Some princes have favored it, from the singularity of its practices; but it is a remarkable circumstance, that in a country where the heads of society are held in immense estimation, it has scarcely been able to make a single proselyte, and its power of propagation has been extinguished by a universal indifference.

At the present time one must search in the recesses of some salons, in order to meet here and there a disciple of Hahnemann. Most frequently they introduce themselves in the wake of an exceptional and extraordinary cure. For us who are accustomed to believe in the remedial powers of nature, we will not deny the greater part of the facts thus loudly proclaimed, but we will reserve to ourselves the right of interpreting them to those who have learnt by observation and study, that which science would lead them to conclude from their investigation. All these men, professors and practitioners, ascribe them to regimen, and the conservative power of the living economy: both of which were perfectly recognized by the ancients, those reasonable and enlightened advocates of the *medicine expectante.*

*Conclusion.*—Homœopathy still exists in Italy, but it is declining, and about to disappear: it has neither professorship nor clinics. Once the ward of an hospital was confided to it at Naples, but it lost it for reasons of impotency well and duly verified. Reduced at the present moment to the treatment of some chronic diseases, which do not require active means, its hopes rest entirely upon the preposessions, the prejudices, or the caprices of persons but little capable of judging it.

*Lond. Med. Gaz.*, Jan. 1845.—From *Med. News.*

